# Spinocerebellar ataxia type 17: Report of a family with reduced penetrance of an unstable Gln_49 _TBP allele, haplotype analysis supporting a founder effect for unstable alleles and comparative analysis of SCA17 genotypes

**DOI:** 10.1186/1471-2350-6-27

**Published:** 2005-07-01

**Authors:** Christine Zühlke, Andreas Dalski, Eberhard Schwinger, Ulrich Finckh

**Affiliations:** 1 Institute of Human Genetics, University of Lübeck, Ratzeburger Allee 160, 23538 Lübeck, Germany; 2 Institute of Human Genetics, Universitätsklinikum Eppendorf, Butenfeld 42, 22529 Hamburg, Germany

## Abstract

**Background:**

Spinocerebellar ataxia type 17 (SCA17), a neurodegenerative disorder in man, is caused by an expanded polymorphic polyglutamine-encoding trinucleotide repeat in the gene for TATA-box binding protein (TBP), a main transcription factor. Observed pathogenic expansions ranged from 43 – 63 glutamine (Gln) codons (Gln_43–63_). Reduced penetrance is known for Gln_43–48 _alleles. In the vast majority of families with SCA17 an expanded CAG repeat interrupted by a CAA CAG CAA element is inherited stably.

**Results:**

Here, we report the first pedigree with a Gln_49 _allele that is a) not interrupted, b) unstable upon transmission, and c) associated with reduced penetrance or very late age of onset. The 76-year-old father of two SCA17 patients carries the Gln_49 _TBP allele but presents without obvious neurological symptoms. His children with Gln_53 _and Gln_52 _developed ataxia at the age of 41 and 50. Haplotype analysis of this and a second family both with uninterrupted expanded and unstable pathological SCA17 alleles revealed a common core genotype not present in the interrupted expansion of an unrelated SCA17 patient. Review of the literature did not present instability in SCA17 families with expanded alleles interrupted by the CAA CAG CAA element.

**Conclusion:**

The presence of a Gln_49 _SCA17 allele in an asymptomatic 76-year-old male reams the discussion of reduced penetrance and genotypes producing very late disease onset. In SCA17, uninterrupted expanded alleles of *TBP *are associated with repeat instability and a common founder haplotype. This suggests for uninterrupted expanded alleles a mutation mechanism and some clinical genetic features distinct from those alleles interrupted by a CAA CAG CAA element.

## Background

The spinocerebellar ataxias (SCAs), a group of autosomal dominantly inherited human disorders with mainly adult age of onset, are caused by progressive neurodegeneration and significant cerebellar dysfunction, but also involve other regions of the central or peripheral nervous system. Clinically and even histopathologically the differentiation between SCA subtypes may be rather difficult. Genetically, for 25 types causative mutations in various genes are known or defined by linkage to distinct chromosomal regions. For seven SCAs expanded CAG trinucleotide repeats have been discovered [1]. This kind of mutation seems to be specific for the human species.

SCA17 [OMIM: 607136], a rare type of SCA with a variety of clinical features, is caused by an expanded CAG repeat in *TBP*, the gene of the TATA box-binding protein [TBP; OMIM: 600075]. TBP forms the DNA-binding subunit of the universally essential RNA polymerase II transcription factor D. TBP has been mapped close to the telomeric region at chromosome 6q27 [2]. An imperfect repetitive and polymorphic polyglutamine encoding CAG triplet sequence is part of the coding region of TBP [3]. Initially, 20 different alleles coding for 29 to 42 glutamine residues (Gln) have been identified [4] with the most common alleles containing 32 to 39 Gln codons. The polyglutamine-encoding DNA sequence of *TBP *wildtype alleles can be subdivided into several regions including two polymorphic (CAG)_n _stretches: (CAG)_3 _(CAA)_3 _(CAG)_n _CAA CAG CAA (CAG)_n _CAA CAG. In patients with SCA17, the polymorphic CAG sequence encoding the polyglutamine stretch of TBP is expanded heterozygous. Alleles with 43 – 48 Gln codons represent a zone of incomplete penetrance or very late age of onset [5,6].

Disease causing alleles with up to 63 [7] Gln codons were identified. Clinically, SCA17 may mimic a broad spectrum of neuropsychiatric diseases, including Parkinson and Huntington disease, major psychosis, and multitudinous cerebellar syndromes [8-10]. Neuropathological findings include cerebellar, cortical, and subcortical atrophy, Purkinje cell loss, gliosis [11], and neuronal intranuclear inclusions immunopositive for TBP and polyglutamine.

The clinical relevance of Gln_43–48 _encoding *TBP *alleles is not obvious. A Gln_43 _allele was considered responsible for clinical symptoms in a 64-year-old patient with ataxia and progressive mental deterioration [6]. Gln_44 _repeats were found in a patient with gait ataxia and behavioral changes and a disease onset at 29 [12] and another patient with early onset cerebellar ataxia (EOCA) and suspected Friedreich ataxia [5]. Regarding the EOCA phenotype in the latter patient, Gln_44 _was considered a large normal allele. On the other hand, alleles for Gln_46 _[12] and Gln_48 _[13] showed reduced penetrance in two families. Therefore, Gln_43–48 _encoding alleles could present intermediate alleles with incomplete penetrance.

In contrast to the majority of other polyglutamine diseases, the SCA17 repeat expansion shows meiotic stability upon transmission. To date, only two pedigrees are known in which instability of the repetitive sequence has been observed, irrespectively of the gender of the transmitting parent. In one case, the repeat increased by one triplet after maternal transmission [5], while in the other case an increase of 13 triplets and marked anticipation was associated with paternal inheritance [14].

Here, we describe a SCA17 family from northern Germany with a) repeat instability upon paternal transmission and b) reduced penetrance or very late onset in association with an uninterrupted CAG_41 _sequence (Gln_49 _allele). Meiotic instability is not common in SCA17 pedigrees. Therefore, we performed haplotype analysis in two unrelated families looking for a founder allele associated with repeat instability at the SCA17 locus. In addition, we included a patient homozygous for a SCA17 allele with a CAG CAA CAG interrupting element to reveal independent mutation events for the different repeat compositions.

## Results

### Phenotype and genotype

In family A, two of four offspring of unaffected parents (74- and 76-year-old) developed an adult onset ataxia. The 43-year-old index patient reported onset of gait disturbances accompanied by a slowly progressive dysarthria at the age of 41. Brain magnetic resonance image analysis performed approximately half a year after clinical onset revealed cortical cerebellar atrophy and a mildly reduced brain volume. His 56-year-old sister noticed symptoms of gait ataxia, dysarthria, and disturbed handwriting at the age of 50. The 76-year-old father of the siblings reported that a neurological examination had revealed no abnormality. Both parents presented without obvious neurological signs. In order to clarify the repeat status of *TBP *and the inheritance of the disease in the family, both agreed in molecular genetic analysis for SCA17 but rejected the offer of a detailed clinical neurological examination.

Molecular genetic analyses revealed pathogenic alleles for the *TBP *gene: 53 repeats for the male patient with age of onset at 41 and 52 repeats for his sister with symptoms starting at 50 years of age. Their clinically non-affected 76-year-old father carries an elongated allele of 49 repeats. Therefore, this allele displays reduced penetrance or very late onset, as well as instability upon transmission. Sequence analysis revealed loss of the CAA CAG CAA interruption in the expanded alleles.

### Haplotype analysis

**Figure 1:**
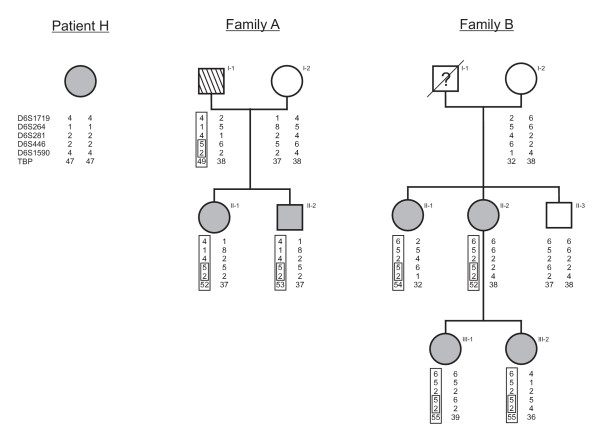
**Pedigrees and haplotype analysis**. Pedigrees of families A and B, including six-locus haplotypes based on five 6q-telomeric microsatellite markers (D6S1719, D6S264, D6S281, D6S446, D6S1590) and closely downstream located polymorphic poly-Gln encoding trinucleotide stretch within *TBP*. Alleles of the microsatellites are denoted by numbers 1 – 8, *TBP *alleles are denoted by the number of Gln-encoding trinucleotides. SCA17-linked alleles are boxed as well as the disease haplotype D6S446 and D6S1590.

Recently, we described a family (B) with repeat instability and missing CAA triplets at the expanded alleles [5,10]. Genealogic data for both families (A,B), as far as available, revealed no relationship between the families. But Northern German ancestry of both families suggested the possibility of a common founder. This was assessed by haplotype analysis (figure 1) including the unrelated patient H carrying homozygous an expansion in the *TBP *gene [15]. In both families A and B, the same haplotype consisting of allele 5 of D6S446 and allele 2 of D6S1590 (5_2) was located on the chromosome bearing the expanded *TBP *allele co-segregating with SCA17, respectively. Patient H did not share this haplotype, she is homozygous for haplotype 2_4. Among the markers analyzed, D6S446 and D6S1590 were the ones most closely located to *TBP*. Therefore, the data suggest the possibility of a common SCA17 founder allele of the variably expanded *TBP *allele in families A and B with further instability upon transmission.

### Allele frequency

To estimate the probability of a founder effect, the allele frequencies of D6S446 and D6S1590 were determined in 96 anonymous control subjects of the geographic region of families A and B. For D6S446 six alleles were found, for D6S1590 seven. Allele 5 of D6S446 is present in 43 out of 184 chromosomes (23%), allele 2 of D6S1590 in 92 (50%). The computed maximum likelihood frequencies (using Arlequin software version 2.001) of the three most common two-locus haplotypes of D6S446 and D6S1590 with frequencies > 0.1 are 0.219 (6_2), 0.163 (6_1), and 0.153 (5_2). Thus, the three most common haplotypes, including the haplotype 5_2 co-segregating with SCA17 in families A and B account for 53.5% of all haplotypes. The 17 remaining haplotypes predicted had computed frequencies from 0.3–6.8%. Maximum likelihood frequency of haplotype 4_2 (patient H) is 0.042. The likelihood ratio test suggested a highly significant linkage disequilibrium between D6S446 and D6S1590 (p < 5 ×10^-6^). Observed and maximum likelihood counts of genotypes and haplotypes did not deviate significantly from Hardy Weinberg equilibrium. Therefore, the likelihood of two unrelated subjects sharing haplotype 5_2 is estimated to be ~8 %. The combined likelihood that two unrelated subjects affected by SCA17 share haplotype 5_2 *and *that this haplotype co-segregates with SCA17 in both families is ~2%. This strongly supports the view of a possible founder haplotype of the SCA17 causing alleles in families A and B.

## Discussion

In comparison to other spinocerebellar ataxias, some genetic features in SCA17 are remarkable: The majority of pathological alleles contains an interspersed CAA CAG CAA element separating the (CAG)_n _sequence into two parts (table 1). In three SCA17 pedigrees known to date the pathogenic allele lacks the CAA CAG CAA interruption and is associated with instability and variable repeat expansion upon transmission. Two of the three families are of northern German origin and were investigated in the study presented. In both families, SCA17 co-segregates with an expanded, uninterrupted, and instable (CAG)_n _in *TBP *located on chromosomes sharing a haplotype in the close centromeric neighborhood of *TBP*, respectively (figure 1). Although the unstable repetitive sequence is linked with one of the three more commonly prevalent haplotypes, statistics point to a nominally significant likelihood that our observation reflects a founder effect rather than coincidence by chance.

In some cases, the expanded glutamine stretch may be the consequence of an intragenic duplication. So, the first published case with SCA17 is a Japanese girl with a de novo duplication event in her paternal allele [7]. In addition, doubling of a stretch of 19 Gln codons was found in a three-generation SCA17 pedigree [16]. Expanded polyglutamine stretches arising from duplications represent rare events in SCAs. Similarly, a pathogenic elongation of the polyalanine part within the polyadenylate binding protein nuclear 1 resulting from duplication has been described for oculopharyngeal muscular dystrophy [17]. The identification of these mutations gives support to the hypothesis of unequal crossing-over as one possible molecular mechanism for repeat expansions. In SCA17, such rare unequal crossing-over events may have been the causative mechanism underlying both the loss of the interspersed CAA CAG CAA element and expansion of (CAG)_n_. As discussed for SCA2 [18] the presence of CAA interruptions in SCA17 alleles breaks the repetitive sequence into shorter homogenous triplet tracts and may thus protect it from instability by reducing the slippage between the complementary strands.

In SCA17, there is a broad range of intermediate *TBP *alleles Gln_43–48 _associated with reduced penetrance. In other SCA types, such intermediate alleles are rarely found [19,20] or even unknown. However, intermediate alleles represent a well-known finding in patients with Huntington disease [21]. We found (CAG/CAA)_49 _but no visible symptoms at the age of 76 in the father of two SCA17 patients with expanded *TBP *alleles (CAG/CAA)_52–53_. This large allele, (CAG/CAA)_49_, has not been found in control samples. It may be of reduced penetrance, associated with very late age of onset and/or low expression of clinical signs. In addition, we performed haplotype analysis for a sporadic patient with homozygosity of the intermediate allele (CAG/CAA)_47 _and a rather progressive course [15]. Here, we cannot exclude the possibility of an additive effect of two intermediate alleles with respect to pathogenicity. In this patient, (CAG/CAA)_47 _is interrupted by CAA CAG CAA and linked with D6S446/D6S1590 haplotype 2_4. Thus, both the type of *TBP *expansion and the SCA17 linked haplotypes differ between the sporadic homozygous and the familial cases presented and do not point to a single founder. An extended analysis of intragenic markers in larger numbers of SCA17 families with different types of expansions could reveal "mutation prone" founder alleles differing with respect to the type of mutation.

## Conclusion

The extraordinary high variability of the clinical expression of pathological *TBP *repeat expansions in SCA17 is further complicated by the occurrence of genetically instable repeats in association with the lack of an interspersing CAA CAG CAA element. The degree of reduced penetrance and very low expression of symptoms refer to strong modifying factors including potent influence of the genetic background. Unless SCA17 is a rare type of dominantly inherited ataxia, the repeat expansion within the *TBP *gene arose at unrelated genotypes. This has to be taken into account both in molecular genetic diagnostics and in genetic counseling.

## Methods

### Molecular analysis and polymorphic markers

Genomic DNA was prepared from peripheral blood leukocytes using standard protocols. SCA17 alleles were amplified by PCR as described [7], separated on 6% denaturing polyacrylamide gels, and visualized by silver staining. For sequencing, PCR products were separated under non-denaturing conditions using the dHPLC-HT-system (WAVE Transgenomic). The eluted fragments were sequenced using dye terminators and the automated capillary sequencer Avant 3100 (Applied Biosystems). For haplotype analysis, the highly polymorphic microsatellite markers D6S1719, D6S264, D6S281, D6S446, and D6S1590 were used as described [15]. Maximum likelihood haplotype frequencies of two-locus haplotypes based on markers D6S446 and D6S1590 genotyped in 96 unrelated anonymous control subjects and linkage disequilibrium were analyzed using Arlequin software version 2.001 [22].

## Competing interests

The author(s) declare that they have no competing interests.

## Authors' contributions

CZ conceived of the study and developed the concept, interpreted the data and contributed substantially to the manuscript (corresponding author). AD carried out the molecular genetic studies. ES participated in the design of the study and helped to draft the manuscript. UF participated in the design and performed the statistical analysis.

All authors read and approved the final manuscript.

## Pre-publication history

The pre-publication history for this paper can be accessed here:



## Figures and Tables

**Table 1:** Review of Gln Repeats and SCA17 Alleles of 16 Unrelated Cases. Duplicated elements are boxed, instability by maternal (m) or paternal (p) inheritance.

**Gln**	**Case**	**Repeat Composition**	**Comments**	**Ref.**
49–53	familial	(CAG)_3 _(CAA)_3 _(CAG)_41–45 _CAA CAG	instability (p)	here
63	sporadic	(CAG)_3 _(CAA)_3 _(CAG)_9 _CAA CAG CAA (CAG)_9 _(CAA)_3 _(CAG)_9 _CAA CAG CAA (CAG)_19 _CAA CAG	duplication	[7]
47	familial	(CAG)_3 _(CAA)_3 _(CAG)_8 _CAA CAG CAA (CAG)_28 _CAA CAG		[16]
47	sporadic	(CAG)_3 _(CAA)_3 _(CAG)_6 _CAA CAG CAA (CAG)_30 _CAA CAG		
48	familial	(CAG)_3 _(CAA)_3 _(CAG)_6 _CAA CAG CAA (CAG)_31 _CAA CAG		
55	familial	(CAG)_3 _(CAA)_3 _(CAG)_9 _CAA CAG CAA (CAG)_16 _CAA CAG CAA (CAG)_16 _CAA CAG	duplication	
51	familial	(CAG)_3 _(CAA)_3 _(CAG)_9 _CAA CAG CAA (CAG)_31 _CAA CAG		[5]
53 – 55	familial	(CAG)_3 _(CAA)_3 _(CAG)_45–47 _CAA CAG	instability (m)	
48	familial	(CAG)_3 _(CAA)_3 _(CAG)_9 _CAA CAG CAA (CAG)_28 _CAA CAG	reduced penetrance	[13]
47	sporadic	(CAG)_3 _(CAA)_3 _(CAG)_9 _CAA CAG CAA (CAG)_27 _CAA CAG	homozygous mutation	[15]
46	familial	(CAG)_3 _(CAA)_3 _(CAG)_9 _CAA CAG CAA (CAG)_26 _CAA CAG		[21]
43		(CAG)_3 _(CAA)_3 _(CAG)_9 _CAA CAG CAA (CAG)_26 _CAA CAG		[6]
53 – 66	familial	(CAG)_3 _(CAA)_4 _(CAG)_44/57 _CAA CAG	instability (p)	[14]
44	sporadic	(CAG)_3 _(CAA)_3 _(CAG)_9 _CAA CAG CAA (CAG)_24 _CAA CAG		[12]
46	sporadic	(CAG)_3 _(CAA)_3 _(CAG)_11 _CAA CAG CAA (CAG)_24 _CAA CAG	reduced penetrance	
48	familial	(CAG)_3 _(CAA)_3 _(CAG)_6 _CAA CAG CAA (CAG)_31 _CAA CAG	homozygous mutation	[23]
